# Acupuncture on the Stress-Related Drug Relapse to Seeking

**DOI:** 10.1155/2018/5367864

**Published:** 2018-10-17

**Authors:** Hyo Sun Roh, Bo Ra Park, Eun Young Jang, Jin Sook Kim, Young S. Gwak

**Affiliations:** ^1^Department of Acupoint, College of Korean Medicine, Dongguk University, Gyeonggi-do 10326, Republic of Korea; ^2^Addiction Research Center and Department of Physiology, College of Korean Medicine, Daegu Haany University, Daegu 42158, Republic of Korea; ^3^Addiction Research Center and Department of Secondary Special Education, Daegu Haany University, Daegu 42158, Republic of Korea; ^4^Department of Anesthesiology, Wake Forest Baptist Medical Center, Winston Salem, NC 27106, USA

## Abstract

Drug addiction is a chronic relapsing disease, which causes serious social and economic problems. The most important trial for the successful treatment of drug addiction is to prevent the high rate of relapse to drug-seeking behaviors. Opponent process as a motivational theory with excessive drug seeking in the negative reinforcement of drug dependence reflects both loss of brain reward system and recruitment of brain stress system. The negative emotional state produced by brain stress system during drug withdrawal might contribute to the intense drug craving and drive drug-seeking behaviors via negative reinforcement mechanisms. Decrease in dopamine neurotransmission in the nucleus accumbens and recruitment of corticotropin-releasing factor in the extended amygdala are hypothesized to be implicated in mediating this motivated behavior. Also, a brain stress response system is hypothesized to increase drug craving and contribute to relapse to drug-seeking behavior during the preoccupation and anticipation stage of dependence caused by the exposure to stress characterized as the nonspecific responses to any demands on the body. Acupuncture has proven to be effective for reducing drug addiction and stress-related psychiatric disorders, such as anxiety and depression. Furthermore, acupuncture has been shown to correct reversible brain malfunctions by regulating drug addiction and stress-related neurotransmitters. Accordingly, it seems reasonable to propose that acupuncture attenuates relapse to drug-seeking behavior through inhibition of stress response. In this review, a brief description of stress in relapse to drug-seeking behavior and the effects of acupuncture were presented.

## 1. Introduction

Acupuncture, as a complementary and alternative therapeutic intervention, has been widely practiced as a therapeutic intervention in eastern countries for thousands of years. Various demonstration of acupuncture including manual, electrical, and laser on acupuncture points have suggested the therapeutic effect of acupuncture on mental and physical disorders [1–3]. Studies in animals and humans have demonstrated that acupuncture caused neurobiological responses in the brain by stimulating specific acupoints on the skin and muscle with insertion of fine acupuncture needles [4].

Acupuncture has been shown to be effective for reducing drug addiction in preclinical and clinical trials [5]. In particular, acupuncture has been accepted to be an effective detoxification method for drug addiction as a standard procedure [6]. Drug addiction is a chronically relapsing disease characterized by uncontrolled or compulsive use of addictive drugs that resulted in serious personal, social, and economic problems [7, 8]. One of the main factors that cause relapses in addicts is the exposure to stress. Stress can be characterized as the nonspecific responses of the body to any demands for a change [9]. Indeed, previous studies suggested that stress-related psychiatric disorders, such as anxiety and depression, commonly led to either drug relapse or the vulnerability to drug abuse [10–12]. Also, addicts displayed stress-induced negative emotions such as fear, sadness, and anger [13]. This suggests that psychological treatment would be an effective therapeutic strategy on the addiction treatment.

Previous studies have suggested that a motivated behavior associated with excessive drug seeking in the negative reinforcement of drug dependence reflects both loss of brain reward function and recruitment of brain stress system. The negative emotional state produced by brain stress system during drug withdrawal might contribute to the intense drug craving and drive drug-seeking behaviors via negative reinforcement mechanisms [14]. Previous studies have shown that acupuncture plays an important role in correcting dysregulation of stress-related brain emotional systems and improves the efficiency of homeostatic mechanisms of emotion [15, 16]. Also, acupuncture has been proven to be an effective treatment for reducing stress, anxiety, and depression via modulation of brain stress and endocrine systems in preclinical and clinical studies [17, 18]. Furthermore, it has been suggested that acupuncture may be an adjunct therapy for the treatment of several mental disorders including drug addiction [19]. Thus, it is not unexpected that acupuncture ameliorates negative emotional states of the addiction cycle by regulating neurotransmitters in the brain that have long been associated with addictive behaviors [20]. Indeed, neurochemical studies have shown that acupuncture reduced not only anxiety-like behavior by normalizing catecholamine levels in the amygdala but also behavioral withdrawal syndromes by normalizing dopamine release in the nucleus accumbens (NAc) in ethanol-dependent rat [21]. Although many methods have been employed in reducing drug addiction, there is currently no satisfactory medical intervention to prevent relapse to drug seeking and there are still many unanswered questions about the basic mechanisms underlying the effectiveness of acupuncture in the treatment of drug relapse, including stress-induced reinstatement. In the present review, we focused on acupuncture's effectiveness in suppressing relapse to drug-seeking behavior by modulating brain stress response systems.

## 2. Neuronal Adaptation on Drug Addiction

Drug addiction has been suggested as a disorder showing impulsivity and compulsivity to seek and take the drug [22]. Previous studies have suggested that opponent process as a motivational theory with excessive drug seeking in the negative reinforcement of drug dependence reflects both loss of brain reward function and recruitment of brain stress system. The negative emotional state produced by brain stress system during drug withdrawal might contribute to the intense drug craving and drive drug-seeking behaviors via negative reinforcement mechanisms [14]. Since the exhilarating rush that reinforces the desire to take drugs occurs from impulsivity to compulsivity, positive reinforcement moves to negative reinforcement in driving the motivated behavior. Drug seeking is triggered by addictive drugs through proponent processes mediated by brain reward system, especially mesolimbic dopamine system, which provides positive reinforcement. The continued drug abuse induced by positive reinforcement causes a new neuroadaptive mechanism that resulted in the dysregulation of the brain reward pathways [22]. This counteradaptive process including the opponent process negatively affects the maintenance or recovery of homeostasis in the brain reward system. Based on an association between drug-taking behaviors and brain motivation, acute effect of a drug itself (positive hedonic effect) was hypothesized to be counteracted by a negative feedback control which led to decreased reward functions via altered set point of reward [23]. Therefore, the counteradaptive process increases drug craving and vulnerability to drug relapse to remove negative emotional state in dependence. This process was hypothesized to produce an altered allostatic state, a predominant neuroadaptive process in drug addiction.

The concept of allostasis was introduced to differentiate the chronic deviation of the homeostasis regulatory system from the normal condition to the newly established set point [24, 25]. This set point was mainly characterized by activation of the hypothalamic-pituitary-adrenal (HPA) axis, the hallmark of the stress reaction [26], and the extrahypothalamic corticotropin-releasing factor (CRF) system of the extended amygdala, a primitive structure of brain stress response systems [27]. Activation of these systems is believed to be responsible for the mechanism, at least in part, underlying negative emotional state including anxiety and depression that accompanies drug withdrawal. Also, this activation contributes to the opponent process as a motivational theory causing relapses to drug-seeking behaviors in the negative reinforcement of drug dependence [14]. Hypofunctional state of reward systems in the opponent process reflects anxiety and anhedonia which accompanies drug withdrawal. These negative motivational states may contribute to vulnerability to relapse during the development of drug addiction [28].

## 3. Effect of CRF and Stress on Drug Relapse

Although the exact neurochemical mechanism mediating stress-induced relapse to drug seeking is not clearly understood, some evidence exists to suggest that stress has been implicated in drug reinstatement. Regarding relapse to drug seeking, mainly two ways exist in chronically sensitizing negative emotional states during acute withdrawal from chronic administration of abused drugs. One way is to develop protracted abstinence and the other way is to increase sensitivity to reinstatement of drug-seeking behavior, as indicated in stress-induced reinstatement [29]. The brain stress hormone, CRF, may be the motivational aspects of drug withdrawal such as ethanol withdrawal. The activation of CRF system in the extended amygdala during acute withdrawal from chronic drug administration may represent the mechanism underlying the opponent process in negative emotional states [30]. Indeed, previous studies have suggested that the brain CRF system plays an important role in mediating anxiety-like behavior and ethanol-seeking behavior in ethanol-dependent rats [31–34].

CRF neurons projecting from the central nucleus of the amygdala to the bed nucleus of the stria terminal mediate the stress-induced relapse to cocaine seeking [35]. Further support for a role of CRF is the significant overlap between the neural circuitry of stress-induced reinstatement and that of acute motivational withdrawal [36, 37]. Literature also demonstrated that the cortical glutamatergic activation contributed to the drug seeking and stress. It is well known that glutamatergic neurotransmission between the PFC and VTA regulate dopaminergic neuronal activity. For example, stress induced the increase of dopamine release in the PFC and NAc, which was attenuated by glutamate receptor inhibition in the VTA [38, 39]. Activation of cortical glutamate neurons has also been implicated in stress-induced relapse to drug-seeking. CRF release in the VTA induced by stress reinstated cocaine seeking through activation of glutamatergic neurons in the VTA and glutamatergic activation in the NAc projecting from prefrontal cortex [40, 41]. Stress also has a significant impact on the mesolimbic dopamine system. Stress reinstated drug-seeking behavior by increasing dopamine release in the NAc, a primitive structure that is responsible for the reinforcing effect of drugs [42], which was blocked by local injection of dopamine receptor antagonist into the NAc [43].

Although not much is known about the neural circuits between the CRF and mesolimbic dopamine system, significant evidence supports an interaction between CRF and dopamine systems in the extended amygdala and the VTA leading to the development of drug dependence and relapse. Drug-withdrawal results in a sustained activation of the CRF system in the extended amygdala. Consequently, the brain CRF system causes the reduction of accumbal dopamine release in drug-dependent and withdrawn subjects [44]. More specifically, chronic hyperactivity of the CRF system produces transient dysfunction of dopaminergic neurons in the brain through oxidative mechanisms. On the other hand, acute stress increases dopamine release in the NAc shell through activation of CRF pathway [45]. In support of this observation, a recent study has shown that exposure to external stressors exacerbates CRF release in the VTA during drug withdrawal in dependent subjects and arouses drug relapse through activation of dopaminergic neurons [46].

## 4. Effect of Acupuncture on Relapse in Drug Dependence

Although few experiments have investigated acupunctural effect on drug relapse, results of animal and clinical studies provided evidence for acupuncture-induced inhibition of drug reward and stress systems in drug dependence. Since the reduction of dopamine levels in the NAc during withdrawal from ethanol administration may be responsible for the negative affect such as anxiety and anhedonia [47, 48], it seems reasonable to propose that acupuncture may reduce the motivated behavior in alcohol dependence by restoring the deficiency in dopamine release in the brain. Previous works have demonstrated that acupuncture at HT7 effectively prevented both the decrease of extracellular dopamine levels in the NAc during ethanol withdrawal and the increase in accumbal dopamine levels induced by the ethanol challenge [49]. In addition, electroacupuncture (EA) attenuated alcohol drinking behavior by enhancing dopamine levels in immobilized rats [50]. Inhibitions of anxiety-like behavior and reductions in tyrosine hydroxylase mRNA expression in the VTA and tyrosine hydroxylase protein expression in the NAc by acupuncture in ethanol-withdrawn rats confirmed these results [51]. Furthermore, the anxiolytic effect of acupuncture was blocked by local infusion of dopamine receptor antagonists SCH23390 (the selective D1 receptor antagonist) and eticlopride (the selective D2 receptor antagonist) into the NAc [52]. Taken together, these findings provide evidence that acupuncture may help to suppress drug relapse and craving by inhibiting dopamine depletion in the mesolimbic dopamine system.

### 4.1. Effect of Acupuncture on BDNF

One study attempted to determine a role for BDNF in the anxiolytic effects of acupuncture. This study showed that acupuncture inhibited anxiety-like behaviors by ameliorating the dysfunction of the mesolimbic dopamine pathway through inhibition of BDNF expression in the VTA in ethanol-dependent rats [51]. However, a role for BDNF in the motivated behavior and the mesolimbic dopamine system in drug dependence still remains unclear. Results of animal studies showed that withdrawal from chronic opiates increased BDNF levels and BDNF-related neuronal plasticity in the VTA and produced aversive withdrawal motivation [53]. Similarly, specific knockdown of viral-mediated BDNF in the mesolimbic dopamine pathway ameliorated social aversion [54, 55]. BDNF levels in the NAc significantly increased in chronic methamphetamine-induced depression-like behavior [56]. Importantly, BDNF has been shown to serve as a key regulator of the mesolimbic dopamine system [57, 58]. Thus, it can be expected that acupuncture can reduce drug relapse by improving negative emotional state in drug dependence through modulation of the BDNF-mediated mesolimbic dopamine pathway. In contrast, it has been shown that repeated electroacupuncture at Baihui (GV-20) and Yintang (EX-HN3) increased glutamate, 5-hydroxytryptamine (5-HT), and the BDNF-related peptides, such as protein kinase C (PKC) and cyclic adenosine monophosphate response element binding protein, in the hippocampus and inhibited stress-induced depression [59]. Although few clinical studies have investigated the effect of acupuncture on brain neurotransmitter levels, it has been shown that acupuncture reduced serum norepinephrine levels and increased serum levels of glutamate, 5-HT, and BDNF in insomnia patients, suggesting that serum BDNF has been implicated in the sedative effect of acupuncture [60, 61].

### 4.2. Effect of Acupuncture on Cannabinoid

Cannabinoid 1 receptor (CB1R) has been suggested to mediate ethanol self-administration by acting on the mesolimbic dopamine pathway [62]. Interestingly, electroacupuncture (EA) attenuated upregulation of CB1R in the striatum, amygdala, and VTA during ethanol withdrawal [63]. Activation of CB1R expressed on VTA GABA neurons inhibited GABAergic transmission and in turn increased dopamine transmission in the NAc [64]. Therefore, it is reasonable to propose that CB1R-mediated inhibition of GABA neurons in the VTA by EA may inhibit hypoexcitability of dopamine neurons in the mesolimbic system during withdrawal from chronic ethanol administration. In addition, cannabinoid agonist HU210 promoted relapse and cocaine-seeking behaviors attenuated by the selective CB1 receptor antagonist SR141716A and inhibition of CB1R attenuated ethanol self-administration and alcohol-seeking behavior [65, 66]. These data revealed an important role of the cannabinoid system in the neuronal processes underlying relapse to cocaine and alcohol seeking and provided a rationale for the use of cannabinoid receptor antagonists for the prevention of relapse to cocaine and alcohol use. As alcoholics expressed CB1R polymorphism and contributed to ethanol dependence [67], EA may reduce alcohol-seeking behavior by modulating CB1R in ethanol-dependent subjects.

### 4.3. Effect of Acupuncture on Opioid

Over a decade, literatures have shown that acupuncture reduced alcohol cravings in alcohol-dependent patients. The microdialysis study raised the possibility that the GABA pathway may mediate acupuncture inhibition of withdrawal syndrome and reduction of dopamine release in the NAc in ethanol-withdrawn rats [49]. In addition, acupuncture decreased ethanol self-administration and suppressed VTA GABA neuronal firing rate through activation of endogenous opioid systems [68]. Previous works have stated that acupuncture may activate *β*-endorphinergic and enkephalinergic neurons in the arcuate nucleus of the hypothalamus [69, 70]. For example, electroacupuncture suppressed morphine withdrawal with a significant increase of brain beta-endorphin level [71]. *β*-Endorphinergic neurons projecting from the arcuate nucleus may trigger opioid receptors on GABA neurons in the VTA and NAc [72]. It is well known that GABA interneurons and GABAergic terminal projected from the NAc synapse with dopamine neurons in the VTA. Therefore, beta-endorphin and endogenous mu-opioid peptides may disinhibit GABA neuronal activity in the NAc and/or VTA that would result in the enhancement of dopamine neuron activity in the VTA, thereby increasing dopamine release in the NAc. Thus, it can be hypothesized that acupuncture may inhibit drug-seeking behavior and craving by normalizing the depletion of dopamine in the NAc via activation of *β*-endorphinergic neurons from the arcuate nucleus of the hypothalamus [73]. Furthermore, it has been suggested that *β*-endorphin in the brain modulated behavioral responses to stress and anxiety in drug-seeking behavior [74]. Although the exact neurochemical mechanism regarding a role for *β*-endorphin is still elusive, some evidence suggests that *β*-endorphin may reduce stress response through inhibition of CRH release [75]. In support of this evidence, pharmacological studies have shown that low frequency EA (2 Hz) inhibited morphine-induced conditioned preference place by increasing enkephalin synthesis in the NAc [76].

### 4.4. Effect of Acupuncture on Stress Response

It is well documented that acupuncture decreases anxiety levels and stress-related symptoms [77, 78]. In addition, studies have demonstrated that acupuncture reduces stress response by the following mechanisms: (1) inhibition of the sympathoadrenal medullary system in rats exposed to immobilization stress [79], (2) inhibition of HPA system in maternal separation rats [80], and (3) suppression of HPA system in ethanol-withdrawn rats [80, 81], respectively.

Many evidences have suggested that the brain stress response system may be an important factor in drug-seeking behavior of drug dependence. Amygdala is a center for the modulation of affective behaviors, such as anxiety, depression, and stress that trigger drug seeking and relapse in drug addiction. Many studies reported that chronic treatment of ethanol, nicotine, or cannabinoid commonly increased CRF levels in the central nucleus of the amygdala whereas CRF antagonists in the amygdala reduced anxiety-like behavior [82–84]. Also, local infusion of CRF receptor antagonist into the central nucleus of the amygdala was reported to inhibit ethanol-seeking behavior in ethanol-withdrawn rats [85]. These suggest that the amygdala may play an important role in mediating stress-induced reinstatement of drug-seeking and anxiety-like response presumably through activation of CRF neurons input to the amygdala. In addition, electroacupuncture attenuated morphine withdrawal and c-Fos expression in the central nucleus of the amygdala and stress-induced CRH level rats [86, 87]. Interestingly, one study has shown that acupuncture inhibited anxiety-like behavior and increases in plasma corticosterone and amygdaloid catecholamine in ethanol-withdrawn rats [85]. Based on CRF-noradrenergic interaction in the amygdala, it was suggested that acupuncture may reduce ethanol withdrawal dysphoric symptoms by modulating the stress response system. Moreover, CRF in the central nucleus of the amygdala has been implicated in the anxiolytic effect of acupuncture in ethanol-withdrawn rat [88]. CRF in the amygdala has been shown to be important in mediating anxiety-like behavior that accompanies ethanol withdrawal and contributed to relapse to ethanol-seeking behavior and drug craving [88]. Therefore, these results raise the possibility that acupuncture may attenuate anxiety-like behavior during ethanol withdrawal through suppression of CRF neurons in the amygdala. Consistent with this finding, acupuncture reduced anxiety-like behavior during nicotine withdrawal by inhibiting CRF expression in the amygdala [89]. These effects have been confirmed in a variety of experimental studies showing that acupuncture suppressed CRF neuronal activity in the various regions of brain including the amygdala [90, 91]. Interestingly, *β*-endorphin is important in reducing response of the hypothalamic CRF neuron to stress and attenuating anxiety-like behavior [92, 93]. Thus, acupuncture inhibition of drug-seeking behavior might result from modulation of stress response system in the amygdala through activation of *β*-endorphinergic neurons in the arcuate nucleus of the hypothalamus.

### 4.5. Acupuncture on Stress-Induced Relapse to Drug Seeking

Multiple types of stimuli trigger craving and subsequent relapse in drug addicts, such as exposure to the drug itself, stress, and drug-associated cues [94–96]. As these stimuli activate dopamine neurons in the VTA followed by increase dopamine release in the NAc, the mesolimbic dopamine system plays an important role in relapse to drug-seeking behavior [97, 98]. More specifically, drug dependents subjected to external stressors revealed increases in drug craving and relapse. This drug relapse induced by stress was suggested to be motivated by self-medication of the associated negative effect of stress [99] and CRF played a key role in mediating stress-induced drug reinstatement [100]. Acupuncture-initiated impulses may activate brain pathways and thus ameliorate the efficiency of homeostatic mechanisms of stress and reward mechanism in the brain [101, 102]. Previous work using cocaine self-administration has shown that acupuncture attenuated stress-induced relapse to cocaine seeking by reducing neuronal activity in the NAc [103]. As acute stress enhances dopamine release in the NAc via activation of CRF neurons [45], this study provided evidence that acupuncture might attenuate relapse to drug seeking by the reduced dopamine response via activation of GABA neurons in the VTA. Theoretically, VTA GABA neurons inhibit the activity of dopamine neurons in the VTA and critically modulate drug reward and relapse [104]. Indeed, activation of VTA GABA neurons by optogenetic stimulation inhibits dopamine neuronal activity in the VTA and in turn decreases dopamine release in the NAc [105]. Interestingly, the recent study showed that acupuncture at Shenmen (HT7) points attenuated cocaine-primed reinstatement, cocaine inhibition of GABA neuron activity in the VTA, and acute cocaine-induced dopamine release in the NAc, which were reversed by local infusion of the selective GABAB receptor antagonist 2-hydroxysaclofen [106]. These results suggest that acupuncture may reduce relapse to cocaine-seeking behavior by enhancement of GABAergic inhibition in the VTA.

Considerable studies have investigated the effect of acupuncture on the mesolimbic dopamine system and relapse to drug-seeking behavior induced by drug, drug-associated cues, and stress. Literature demonstrated that EA reduced cue-evoked heroin-seeking and reinstatement of heroin-seeking by heroin priming and extinction responding of heroin-seeking behavior and Fos expression in the NAc core [105, 107]. In addition, the functional MRI (fMRI) study has shown that acupuncture inhibited cue-induced heroin craving and brain activation [108]. These results suggest the possibility that acupuncture may reduce discrete cue-induced drug-seeking behavior by modulating dopamine release in the NAc. Also, it has been shown that acupuncture reduced morphine or cue-induced reinstatement of morphine-seeking behaviors and morphine craving in progressive ratio via activation of GABA receptors [109, 110]. Similarly, acupuncture decreased extracellular dopamine levels in the NAc and behavioral sensitization induced by repeated morphine administration [111]. Given that drug sensitization may induce relapse to abused drugs, it can be suggested that acupuncture may reduce morphine-seeking behavior. This suggestion is supported by the finding that acupuncture inhibited behavioral sensitization induced by repeated cocaine administration via modulation of dopamine neuron activity in the VTA [112].

## 5. Acupuncture in Clinical Therapy

Over a decade, the clinical applications of acupuncture to the prevention or attenuation of drug craving or relapse have been suggested [113, 114]. Clinical studies have shown that chronic acupuncture [twice a week for weeks, Zhubin (KI9) acupoint)] reduced alcohol craving in alcohol-dependent patients using measures of the Visual Analogue Scale (VAS) [18] and transcutaneous electrical acupoint stimulation (TEAS, for 3 months) decreased the relapse rate of rehabilitating heroin user [115]. Acupuncture attenuated both ethanol intake and relapse in alcoholics [116, 117]. Acupuncture also has proven to be an effective treatment for reducing stress, anxiety, and depression via modulation of brain stress and endocrine systems in clinical studies [118]. The fMRI studies reported that acupuncture increased the self-alcohol abstinence in social alcohol drinkers and activated brain areas that have been associated with self-control over alcohol craving [119] and reduced craving by smoking-related visual cues [120]. Other studies have reported that electroacupuncture suppressed the relapse rate and plasma *β*-endorphin and dynorphin A levels in heroin addicts and the prolonged acupuncture treatment showed dominant control effects on heroin craving behaviors with little side effects [121, 122]. Importantly, acupuncture's role has been reviewed in treating drug dependence in drug-dependent patients including opioid, nicotine, cocaine, and alcohol [123]. Interestingly, the recent fMRI studies have suggested that acupuncture improved depressive behaviors in depressive patients through modulation of limbic system especially amygdala and subgenual anterior cingulate cortex [124] and the corticostriatal reward circuitry [125]. Based on an association between depression and drug relapse in dependence, these data raise the possibility for acupuncture's role in suppressing drug relapse. Taken together, these clinical studies provide evidence that acupuncture may be an effective therapy for reducing drug relapse to drug-seeking behaviors.

## 6. Summary

There is a lot of interest in gaining a better understanding of how acupuncture works in the brain related to addictive behaviors. There are numerous studies in laboratory animals and humans in the field of acupuncture and drug addiction, and such researches have contributed to the significant improvement in determining the basic mechanisms of acupuncture in the treatment of drug addiction. However, the fact that a significant stress gives an excuse for the compulsive drug use and loss of control over drug is an important factor to elucidate the biological mechanism of drug addiction. This stress-induced psychological positive anticipation triggers and facilitates relapse to drug-seeking behaviors. Also, dysregulation of brain reward mechanisms is believed to involve the mesolimbic dopamine system and the brain stress system in some way.

In conclusion, studies provide strong evidences that acupuncture reduces stress-induced negative emotional state and relapse to drug-seeking behavior by modulating the mesolimbic dopamine system and CRF stress system. A better understanding of acupuncture's role may lead to the development of successful therapeutic intervention in the treatment of drug addiction associated with stress.
